# Risk factors for bovine mastitis with the isolation and identification of *Streptococcus agalactiae* from farms in and around Haramaya district, eastern Ethiopia

**DOI:** 10.1007/s11250-019-01838-w

**Published:** 2019-02-11

**Authors:** Biniam Tsegaye Lakew, Taresa Fayera, Yimer Muktar Ali

**Affiliations:** 1 College of Veterinary Medicine, Haramaya University, P.O.Box 138, Dire Dawa, Ethiopia; 2 School of Environmental and Rural Science, University of New England, Armidale, 2351 Australia

**Keywords:** CMT, Haramaya district, Risk factors, *Streptococcus agalactiae*

## Abstract

A cross-sectional study was carried out to determine the prevalence and risk factors of bovine mastitis caused by *Streptococcus agalactiae* from farms in and around Haramaya district, eastern Ethiopia. A total of 384 lactating cows were selected from small-, medium-, and large-scale production systems. California mastitis test (CMT) was used for screening subclinical mastitis. Out of the total animals examined, 63.02% (*n* = 242) had mastitis, where 6.77% (*n* = 26) and 56.25% (*n* = 216) were clinical and subclinical mastitis respectively. The quarter-level prevalence was 29.04% (*n* = 446), from which the clinical form was 6.38% (*n* = 98) and the subclinical was 22.66% (*n* = 348), and the rest quarters were blind 3.32% (*n* = 51). Milk samples from clinical as well as CMT positive quarters were cultured for isolation of *S. agalactiae*, where 10.3% (*n* = 46) resulted in growth of the bacterium. The prevalence of mastitis was found to be statistically significant among the age groups (*p* = 0.002), breed (*p* = 0.000), and parity (*p* = 0.000). Similar findings were found to the extrinsic risk factors considered; as production type (*p* = 0.010), teat injury (*p* = 0.02), and type of floor (*p* = 0.000). The study confirmed the importance of *S. agalactiae* as the cause of contagious mastitis and also identified the associated risk factors in the study farms and hence warrants serious attention.

## Introduction

Mastitis is the inflammation of the mammary gland that has over 130 different isolated causative agents from mastitis milk samples but *Staphylococcus aureus*, *Streptococci*, and members of the *Enterobacteriaceae* are among the most common etiological agents in cows and in other animal species (Quinn et al. 1999). It is often classified as subclinical or clinical depending on the severity of the disease or contagious and environmental based on the causative agents (Quinn et al. 2002; Andrews et al. 2003). Mastitis caused by *Staphylococcal* and *streptococcal* are the commonest and economically a great concern for dairy farming. Unlike *Staphylococcus aureus*, *Streptococcus agalactiae* is one of the mastitis-causing bacteria that can only grow and multiply in the udder (Andersen et al. 2003). However, it can survive for short time periods on hands, milking machine parts and teat skin, leading to its spread from cow to cow during milking. *S. agalactiae* is most commonly introduced into a clean herd when an infected cow is purchased. Because of the silent nature of infections and highly contagious nature, infections can spread quickly (Sandy 2011). As with most infectious diseases, mastitis risk factors depends upon three components; exposure to microbes, cow defense mechanisms, and environmental and management factors (Mungube et al. 2004).

Mastitis has been contributing to reduced milk production and a major source of economic loss to the dairy industry (Erskine 1992), through reduced milk yield and quality, cost of drugs and veterinary treatment, discarded milk, and forced culling (Quinn et al. 1999). Mungube et al. (2005) estimated the economic losses from urban and peri urban areas of Addis Ababa, to be US$58 and 78.65 per cow and per lactation, respectively. In addition to its economic impact, *Streptococcus agalactiae*; group B Streptococcus (GBS), is the major etiologic agent of invasive neonatal infections in humans in industrialized countries, causing sepsis, pneumonia, meningitis, Osteomylits, and soft tissue infections (Baker 2000).

In Ethiopia, a few studies have been conducted with the purpose of estimating the prevalence of bovine mastitis (Kifle and Tadele 2008; Almaw et al. 2009; Sori et al. 2011; Dabash et al. 2014). However, mastitis as a disease particularly the subclinical mastitis has received very little attention. Therefore, the study was conducted with the objectives to determine the prevalence and associated risk factors of bovine mastitis caused by *Streptococcus agalactiae* from farms in and around Haramaya district, Ethiopia.

## Materials and methods

### Study areas

The study was conducted in selected small holder, medium- and large-scale dairy farms in and around Haramaya district, Ethiopia. It is located 503 km east of Addis Ababa; at 41°59′58″ latitude and 09°10′24″ longitudes with 2000 m a.s.l. The district receives an average annual rain fall approximately 900 mm, and climatically, there are two ecological zones of which 66.5% is midland and 33.5% is lowland (Shimelis 2010).

### Study population and husbandry practice

Lactating Holstein-Zebu and local Zebu breeds from 20 dairy farms in and around Haramaya district were categorized into small-scale dairy production (SSDP), medium-scale dairy production (MSDP), and large-scale dairy production (LSDP) based on herd size having 5 or less, 6–30, and 72–171 dairy cattle, respectively (Mureda and Mekuria 2008). The cows in Haramaya University dairy farm were all cross breeds (Holstein Friesian × Zebu) and milked by a milking machine twice a day (morning and afternoon) in a separate milking parlor. The cows were managed under intensive husbandry practice in stall barn made of concrete floor. They were mainly fed hay, brans, and silage. Regular washing of milker’s hand before and after milking of the cows is an established practice at the farm. Age of animals was determined from birth records and categorized as young adults (3–6 years), adults (6 to ≤ 10 years), and old (> 10). Stage of lactation was categorized as early (1–4 month), middle (> 4–8 month), and late (> 8 month to the beginning of dry period). Parity was categorized as few (with ≤ 3calves), moderate (4–7 calves), and many (> 7 calves) (Biffa et al. 2005). The barn floor was grouped into poor (barn which was not well managed and muddy) and good (barn floor which is concrete or well managed)**.**

### Sample size determination

The desired sample size for the study was calculated using the formula given by Thursfield (2005) with an expected prevalence rate of 50%, 95% confidence interval, and 5% absolute precision:

$$ \frac{n={1.96}^{2\ast }{p}_{\mathrm{exp}}\left(1-{p}_{\mathrm{exp}}\right)}{d^2} $$

where

*n*: required sample size
*p*_*exp*_: expected prevalence
*d*^2^: desired absolute precision

So, a total of 384 lactating cows with about 1485 teat quarters were considered for the study.

### Study design and sampling strategy

A cross-sectional study was conducted to determine the prevalence and associated risk factors of bovine mastitis caused by *S. agalactiae.* Cows were examined directly for clinical and indirectly using CMT for subclinical mastitis. Purposive sampling method was used to select study farms based on their willingness to be part of the study. The study animals were only lactating cows and were selected randomly.

### Study methodology

#### Structured questionnaire

Structured questionnaires were developed to include information on cow attributes such as breed, age, parity number, lactation stage, teat or udder condition (lesion, fibrosis, atrophy), tick infestation of udder or teat, presence of blind teat, milk condition (watery, bloody, pussy). The age, lactation stage, and parity numbers were recorded from farm record documents, farm owners, and milkers. The farm attributes like herd size, production type, and status of barn floor were also considered in the questionnaire.

#### Clinical inspection and preparation of udder and teat for sample collection

First, udders and teats were physically examined by visualization and then palpation to detect if there is fibrosis, visible injury, tick infestation, atrophy of tissue, and any blindness. The udder and teats were disinfected with alcohol impregnated cotton, and washing is practiced when the udder is full of dung or dirty materials. The teat on the far side of the udder is cleaned first than those on the near side. Scrubbing was continued until the towel remains clean (Moges et al. 2011).

#### Milk sample collection and CMT

The first two streams of milk were discarded and approximately 2–3 ml of milk samples was collected into the mastitis paddle from individual quarters immediately after the udder is dry. Teats towards sample collection were taken first and then far once (Christos 2011). CMT was carried out on all sample collected in the mastitis paddle. The CMT reagent is mixed with the quarter milk sample that has been collected in the mastitis paddle in approximately equal proportion of the milk sample. Then, after the mixture was swirled in rotary motion, the result is then read within 10–15 s as negative, trace, + 1,+ 2, and + 3 (Radostitis et al. 2007).

#### Bacteriological isolation and characterization

Approximately 10 ml of milk from positive quarters collected into sterile test tubes was placed in ice box and transported to the Haramaya University, Veterinary Microbiology Laboratory. The milk samples were bacteriologically examined according to the procedures employed by Quinn et al. (1999). A loopful of milk sample was streaked on blood agar base enriched with 7% sterile sheep blood for each quarter. Blood agar plates were incubated aerobically at 37 °C for 24–48 h. The plates were examined for gross colony morphology, Gram’s stain, and hemolytic characteristics after 24–48 h. Presumptive colonies of *Streptococcus* species were selected and sub-cultured on nutrient agar and Edward media and incubated aerobically at 37 °C for 24–48 h. The catalase negative cocci were considered as Streptococci (Quinn 2002). The esculin negative colonies were preserved on nutrient agar plates for CAMP (Christie, Atkins, Munch-Petersen) test. *S. agalactiae* were identified by the hemolysis, not hydrolyzing esculin on Edward media and CAMP test.

### Data management and analysis

The data generated during the sample collection and from the questionnaire were entered into the Microsoft Excel spread sheets and was later analyzed by using STATA version 11 software. The effect of risk factors with possible association of the disease was analyzed using chi-square. The associations between dependent and independent variables were tested, and *p* < 0.05 was taken as statistically significant.

## Results

### Overall prevalence

Out of 384 lactating cows, 63.02% (*n* **=** 242**)** were affected by mastitis and from the total of 1536 quarters examined, the prevalence to the quarter level was 29.04% (*n* = 446) and the rest 3.32% (*n* = 51) were blind. The prevalence of both clinical and subclinical at quarter and cow level is shown (Table 1).

**Table 1:** The prevalence of bovine mastitis at cow and quarter levels

	Cow level		Quarter level		
	Positives	Prevalence (%)	No. of teats	Positives	Prevalence (%)
Blind	51	13.28	1536	51	3.32
Clinical	26	6.77	1536	98	6.38
Subclinical	216	56.25	1536	348	22.66
Total	242	63.02	1536	446	29.04

### Isolation of *Streptococcus agalactiae*

**Fig. 1:**
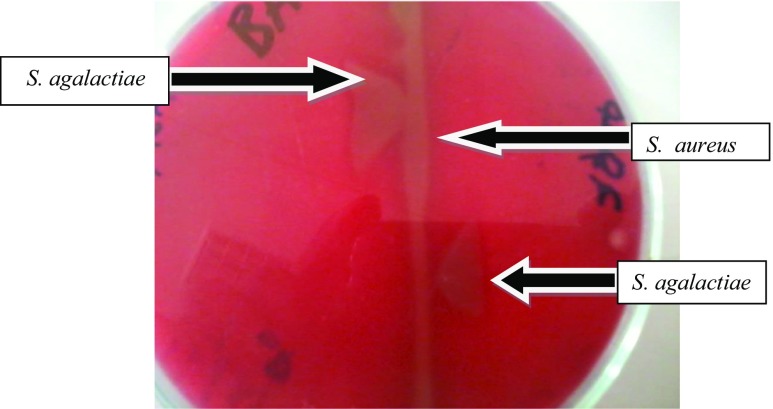
The “arrow head” formation at the junction of *Staphylococcus aureus* and *Streptococcus agalactiae*

A total of 446 milk samples were collected and cultured from clinically and CMT-positive quarters; the prevalence of the *S. agalactiae* at the quarter level was found to be 10.3% (Fig. 1).

### Prevalence based on risk factors

#### Intrinsic risk factors

The prevalence of mastitis at the cow level showed statistically significant difference (*p* < 0.05) among different parity numbers, breeds, and ages considered in the study (Table 2).

**Table 2:** The prevalence of mastitis based on the intrinsic risk factors

Intrinsic risk factors	Total animals examined	Positive animals (%)	prevalence	χ^2^	*p* value
Breed					
Local	74	29(39.2)	7.55	23.3	0.000
Crossbreed	310	213(68.7)	55.47		
Age					
Young adult	96	50 (52.1)	13.02	17.3	0.002
Adult	137	78 (56.9)	20.31		
Old	151	114 (75.5)	29.69		
Parity					
Few	201	89 (44.3)	23.18		
Moderate	65	40 (61.5)	10.42	88.1	0.000
Many	118	113 (95.8)	29.43		
lactation stage					
Early	206	135 (65.5)	35.16	6.2	0.185
Middle	95	50 (52.6)	13.02		
Late	83	57 (68.7)	14.84		
Total	384	242	63.02		

#### Extrinsic risk factors

Management factors such as production type, teat injury, and floor types were also evaluated where all of them had a statistically significant difference (*P* < 0.05) on the prevalence of mastitis (Table 3).

**Table 3:** The prevalence of bovine mastitis based on the extrinsic risk factors

Extrinsic risk factors	Total animals examined	Positive animals (%)	prevalence	χ^2^	*p* value
Production types					
Small scale	39	18 (46.2)	4.69	13.4	0.010
Medium scale	177	103 (58.2)	26.82		
Large scale	168	121 (72.0)	31.51		
Teat injury					
Present	11	10 (90.9)	2.60	7.9	0.02
Absent	373	232 (62.2)	60.42		
Tick infestation					
Absent (negligible)	363	226 (62.3)	58.85	3.85	0.427
Moderate	7	4 (57.1)	1.04		
Infested	14	12 (85.7)	3.13		
Type of floor					
Concrete	306	209 (68.3)	54.43	18.58	0.000
Muddy	78	33 (42.3)	8.59		
Milking type					
Manual	328	201 (61.3)	52.34	3.6	0.165
Machine	56	41 (73.2)	10.68		
Total	384	242	63.02		

### Prevalence of bovine mastitis at farms level

The study also revealed the prevalence of mastitis at the different farms (Table 4).

**Table 4:** The prevalence of mastitis at farm level

Farms	Total animal examined	Clinical (%)	Prevalence (%)	Subclinical (%)	Prevalence (%)	Total (%)
1	38	4 (10.53)	1.04	23 (60.5)	5.99	27 (7.03)
2	14	3 (21.43)	0.78	7 (50)	1.82	10 (2.6)
3	22	1 (4.55)	0.26	8 (36.36)	2.08	9 (2.34)
4	5	0	0	4 (80)	1.04	4 (1.04)
5	5	1 (20)	0.26	3 (60)	0.78	4 (1.04)
6	23	0	0	13 (56.5)	3.39	13 (3.39)
7	3	0	0	2 (66.7)	0.52	2 (0.52)
8	12	1(8.33)	0.26	3 (25)	0.78	4 (1.04)
9	3	1 (33.33)	0.26	0	0	1 (0.26)
10	5	1 (20)	0.26	0	0	1(0.26)
11	4	0	0	0	0	0
12	5	0	0	2 (40)	0.52	2 (0.52)
13	23	1 (4.35)	0.26	14 (60.9)	3.65	15 (3.91)
14	4	0	0	3 (75)	0.78	3 (0.78)
15	5	0	0	3 (60)	0.78	3 (0.78)
16	56	3 (5.36)	0.78	38 (67.9)	9.9	41 (10.68)
17	29	0	0	18 (62.1)	4.69	18 (4.69)
18	27	2 (7.4)	0.52	16(59.25)	4.17	18 (4.69)
19	27	1 (3.7)	0.26	13 (48.15)	3.39	14 (3.65)
20	74	7 (9.46)	1.82	46 (62.16)	11.98	53 (13.8)
Total	384	26	6.77	216	56.25	242 (63.02)

## Discussion

The study showed that the prevalence of bovine mastitis from farms in and around Haramaya district to be 63.02% at cows’ level as determined by the CMT and clinical examinations of the udder. This finding is in agreement with the report of 63.11% by Kassa et al. (2014) in Hawassa and Wando Genet and 61.11% by Tolla (1996) in South Wollo. However, the prevalence was higher than the report of 34.9% by Biffa et al. (2005), 40.40% by Dego and Tareke (2003), 52.9% by G/Michael et al. (2013) in Southern Ethiopia, and 46.7% by Abera et al. (2013) in Adama town and 53.25%; by Biniam et al. (2015) in Dire Dawa town but lower than the report of Mekibib et al. (2010a, b) in Holeta town in Central Ethiopia and Zeryehun et al. (2013) in and around Addis Ababa who reported 71.05 and 74.7% respectively. This variability in the prevalence between different reports could suggest the complexity of the disease, which involves the interaction of several factors mainly of farm management practices, production type and environment, animal risk factors, and causative agent; its prevalence is expected to vary from place to place (Radostitis et al. 2007).

The prevalence of clinical and subclinical mastitis were 6.77 and 56.25%, respectively. The clinical prevalence in this study was comparable to the report of Bishi (1998) who reported the prevalence of 5.3% in Addis Ababa and lower than those reported by Tolosa et al. (2009) who reported the prevalence of 9.5% at Wolayta Sodo and Hundera et al. (2005) with the prevalence of 16.11% in and around Sebeta. In case of subclinical mastitis, the prevalence at cow level (56.25%) in this study was comparable with the finding 54.4% reported by Biffa et al. (2005), 55.1% by Zeryehun et al. (2013), and 55.8% by Bedada and Hiko (2011) but higher than 36.67% reported by Sori et al. (2005) and 44.16% by Biniam et al. (2015). The overall prevalence of subclinical mastitis at both cow and quarter level was found to be higher than clinical mastitis. This could be attributed to the little attention given to subclinical mastitis while treating clinical cases. According to Sori et al. (2005), subclinical mastitis was higher than clinical mastitis owing to the defense mechanism of the udder, which reduces the severity of the disease. Moreover, farmers in Ethiopia are not well informed about the silent cases of mastitis (Zeryehun et al. 2013).

The prevalence of mastitis was higher in older cows (29.69) than young adults (13.02%) and adults (20.31%). The increasing prevalence of mastitis with increasing age is in agreement with the findings by Dego and Tareke (2003) and by Abera et al. (2013) who found that the risk of mastitis increase significantly with the advancing age of the cow. Radostitis et al. (2007) have explained that older cows have largest teats and more relaxed sphincter muscles, which increase the accessibility of infectious agent in the cows’ udder. The increase in prevalence of mastitis with parity reported in the study is comparable with the previous reports (Biffa et al. 2005; Tamirat 2007; Mekibib et al. 2010a, b; Moges et al. 2011; Biniam et al. 2015). This might be due to the increased opportunity of infection with time and the prolonged duration of infection, especially in a herd without mastitis control program and also an increase for teat injuries (Radostitis et al. 2007).

The study also showed that there were significant statistical association between prevalence of mastitis with herd size, floor types, and breeds. This finding is in agreement with Sori et al. (2005); Moges et al. (2011); Kassa et al. (2014). Quinn et al. (1999) have explained that genetic predisposition factors to mastitis such as teat shape, sphincter tone, anatomy of the teat canal, and susceptibility to weakening of the suspensory ligament (“pendulous udder”). In line with this, it was found in this study that the prevalence of mastitis in crossbred cows was statistically higher than that of local cattle.

From 446 milk samples subjected to bacteriological examinations, 10.3% (*n* = 46) of *S. agalactiae* was isolated. This finding is comparable with the report of Zeryehun et al. (2013) and Yohannes and Molla (2013) which were 21.2 and 17.78% respectively and much higher than the report of G/Michael et al. (2013) which was 1.6%, but lower than 26.5% by Megersa et al. (2012). A high proportion of *S. agalactiae* (17.36%) was isolated from CMT-positive cows. This could be because *S. agalactiae* is a highly contagious obligate parasite of the bovine mammary gland (Meiri-Bendek et al. 2002).

## Conclusion

The study showed that the prevalence of mastitis at cow and quarter levels to be high which affects the dairy production. In this study, *S. agalactiae* was isolated more from subclinically infected cows. This indicates that contagious mastitis was prevailing in the studied farms and could be associated with unhygienic milking practice and poor herd management by the farms.
